# Individuality embedded in the isolation calls of captive beluga whales (*Delphinapterus leucas*)

**DOI:** 10.1186/s40851-015-0028-x

**Published:** 2015-10-01

**Authors:** Yuka Mishima, Tadamichi Morisaka, Miho Itoh, Ikuo Matsuo, Aiko Sakaguchi, Yoshinori Miyamoto

**Affiliations:** The Graduate School of Marine Science and Technology, Tokyo University of Marine Science and Technology, 4-5-7, Konan, Minato-ku, Tokyo, 108-8477 Japan; Institute of Innovative Science and Technology, Tokai University, 3-20-1, Orido, Shimizu-ku, Shizuoka-shi, Shizuoka 424-8610 Japan; Port of Nagoya Public Aquarium, 1-3, Minato-machi, Minato-ku, Nagoya, Aichi 455-0003 Japan; Department of Information Science, Tohoku Gakuin University, 2-1-1, Tenjinzawa, Izumiku, Sendai, Miyagi 981-3193 Japan

## Abstract

**Introduction:**

Species with fission-fusion social systems tend to exchange individualized contact calls to maintain group cohesion. Signature whistles by bottlenose dolphins are unique compared to the contact calls of other non-human animals in that they include identity information independent of voice cues. Further, dolphins copy the signatures of conspecifics and use them to label specific individuals. Increasing our knowledge of the contact calls of other cetaceans that have a fluid social structure may thus help us better understand the evolutionary and adaptive significance of all forms of individually distinctive calls. It was recently reported that one type of broadband pulsed sounds (PS1), rather than whistles, may function as individualized contact calls in captive belugas. The objective of this study was to assess the function and individual distinctiveness of PS1 calls in an isolation context. Recordings were made from five captive belugas, including both sexes and various ages.

**Results:**

PS1 was the predominant call type (38 % in total) out of five broader sound categories. One sub-adult and three adults had individually distinctive and stereotyped pulse repetition pattern in PS1; one calf showed no clear stereotyped pulse repetition pattern. While visual inspection of the PS1 power spectra uncovered no apparent individual specificity, statistical analyses revealed that both temporal and spectral parameters had inter-individual differences and that there was greater inter-individual than intra-individual variability. Discriminant function analysis based on five temporal and spectral parameters classified PS1 calls into individuals with an overall correct classification rate of 80.5 %, and the most informative parameter was the average Inter-pulse interval, followed by peak frequency.

**Conclusion:**

These results suggest that belugas use individually distinctive contact calls in an isolation context. If belugas encode signature information in PS1 calls, as seen in bottlenose dolphins, the pulse repetition pattern may be the carrier, as it is individually stereotyped and appears to require vocal development. This idea is supported by the finding that the average inter-pulse interval is the most powerful discriminator in discriminant analysis. Playback experiments will elucidate which parameters are perceived as individual characteristics, and whether one of the parameters functions as a signature.

**Electronic supplementary material:**

The online version of this article (doi:10.1186/s40851-015-0028-x) contains supplementary material, which is available to authorized users.

## Introduction

Group-living animals need to contact each other in order to maintain group cohesion, and one effective means of this is the exchange of vocal signals referred to as “contact calls.” Contact calls have species-specific features, and function as signals for inter-individual cohesion or movement coordination within groups, as well as identity advertisement [1]. The type of identity information in contact calls is linked to social structure. Typically, socially stable species have group-specific contact calls that are shared by group members, and species with fission-fusion social systems have individual-specific contact calls [2].

The individuality of contact calls is seen in birds [2] and mammals, including elephants [3], bats [4], primates [5], and cetaceans [6]. Bottlenose dolphins *(Tursiops truncatus*) are the most extensively studied cetacean species. They live in fluid fission-fusion societies, and have individualized contact calls called “signature whistles” [6, 7]. Early studies defined the signature whistle as the dominant whistle type in an isolation context [7, 8], and this definition has been used in subsequent studies [9–11]. Signature whistles are characterized by low-frequency tonal sounds from 1 to 30 kHz with or without harmonics, and 0.1–4.0 s in duration [12]. Signature whistles are different from contact calls in other animals. One conspicuous peculiarity of signature whistles is that individual identity information is encoded in the frequency contour independent of the voice feature [13], the latter of which is a by-product of individual differences in the vocal tract and body size, as is seen in most other species [14]. During the first year of life, bottlenose dolphins develop their own novel signature whistles via production learning [15, 16]. Dolphins often exchange signature whistles within a 1-s interval [17, 18], but they copy the signature whistles of close associates at lower rates [10, 17–19]. This copying is then used to address a specific animal [20, 21]. Those signature roles require complex cognitive abilities, such as vocal production learning, the ability to copy novel sounds (copying is differentiated from production learning in that it is not clear whether the copying is derived from acquisition of new production, i.e., production learning, or crystallization of originally possessed signals), and the ability to label specific objects using sounds. Signature whistles are thus exceptional among other contact calls in non-human animals. Other than the names of humans, the closest mechanism to the signature whistles of bottlenose dolphins has been identified in the contact calls of orange-fronted conures (*Aratinga canicularis*), which also have individually distinctive frequency contours in contact calls [2], and copy other associates’ contact calls to address specific individuals in fission-fusion flocks [22]. In the present study, we asked whether this pattern is unique to these species, and specifically whether other cetaceans produce these types of contact calls.

Beluga whales (*Delphinapterus leucas*) are a circumpolar and annual-migratory species. They migrate long distances from overwintering areas in polynyas to summering areas in coastal and adjacent offshore waters [23–27]. Their group composition is mainly separated into two types: 1) matrilineal groups of females, calves, and juveniles and 2) smaller groups of males [24]. Matrilineal groups maintain associations with their group members during migration [25]. Mothers and calves remain together during the summer, but other members tend to associate with non-relatives. Moreover, several groups of related belugas may intermingle in the same areas, as is evidenced by the lack of associations other than mother-calf pairs when sampled in the summering areas [25]. Adult males appear to disperse from their matrilineal groups and form long-term social bands with other mature males [24, 25]. Their high mobility, fluid fission-fusion social structure, and long-term associations suggest that this species may have individually distinctive contact calls. In addition, given their potential production learning during vocal development [28], their ability to copy human speech and synthetic sounds [29, 30], and object labeling skills using sounds [31], there is a clear possibility that these whales may integrate these skills into an individual recognition system of contact calls akin to that in bottlenose dolphins. Belugas are highly vociferous and generate a variety of calls using tonal and pulsed components [32–42], and they are often referred to as “sea canaries.” Although little is known about contact calls in belugas, they likely use pulsed sounds rather than whistles for contact [28, 35, 39, 41, 43]. Van Parijis (2003) reported that a temporarily captured mother-calf pair produced pulsed sounds [35]. Vergara and Barrett-Lennard (2008) and Vergara et al. (2010) also revealed that one type of broadband pulsed sound, “Type A” calls, functioned as mother-calf contact calls [28, 39]. The Type A variants, which differed in pulse repetition rate and energy distribution, did not convey individual identity. However, the possibility that each variant could exhibit identity coding based on some parameters, even if each particular variant per se is not an individual signature, remains unexplored. Previously, Morisaka et al. (2013) suggested individuality in the contact calls of belugas [43]. They found that belugas frequently exchanged one type of broadband pulsed sound (“PS1”), and the vocal exchange patterns of PS1 calls resembled those of signature whistles in bottlenose dolphins [18]. Further, the pulse repetition patterns of PS1 calls differed among three adults. PS1 thus appears to function as an individualized contact call, but only a small amount of data was analyzed in a short period. Recordings were made in various contexts, so the data may include context effects on the call rate or parameters. In addition, the Morisaka study only focused on the temporal domain for individual comparisons.

In the present study, we conducted a subsequent investigation of PS1 calls to evaluate their signature function. This is the first step in assessing whether PS1 calls have a parallel function to signature whistles. Recordings were conducted on each of five captive belugas, including both sexes and various ages, in an isolation context. As described above, isolation contexts have been widely used in bottlenose dolphin studies to identify individual signature whistles [7–11], and it is a suitable condition in which contact calls are elicited from both the isolated animal and other remaining associates. Therefore, an isolation context, similar to the conditions used to define signature whistles in bottlenose dolphins**,** was provided for each beluga (with the exception of the mother and the calf). PS1 calls from a calf were also collected in the context in which he was separated from other group members with his mother or another sub-adult. We examined the individuality of PS1 calls using temporal and spectral parameters.

## Materials and methods

### Facility and subjects

**Fig. 1:**
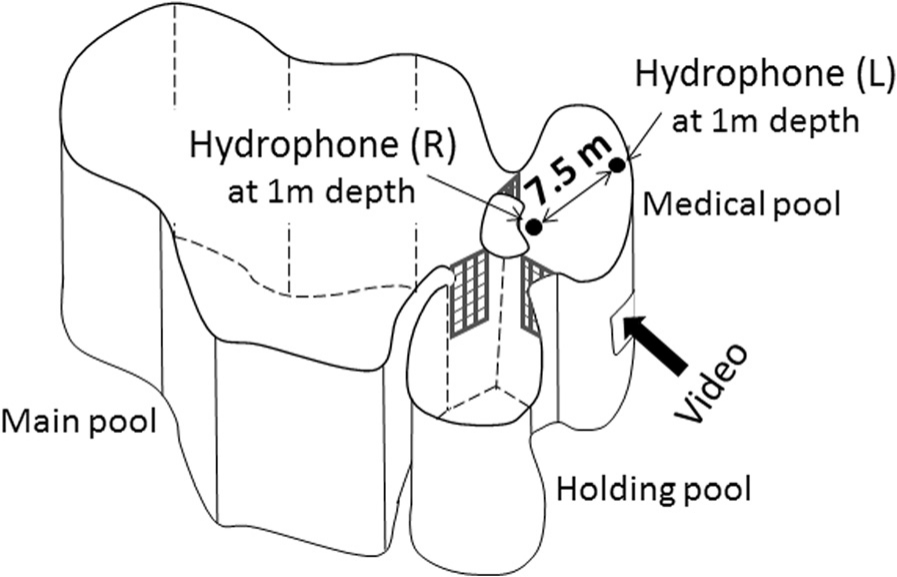
Schematic view of the beluga pool in the Port of Nagoya Public Aquarium, Japan. Each of the belugas was isolated in the medical pool

Data were collected at the Port of Nagoya Public Aquarium in Nagoya City, Aichi, Japan from September 2013 to May 2014. There were five belugas: one adult male (H), two adult females (T, G), one sub-adult female (N), and one male calf (M). H came from the White Sea, Russia in 2001, and its estimated age was 36 years old. T and G came from the Russian Far East in 2001, and were estimated to be 19 and 15 years old, respectively. N and M were born at the aquarium. N is the daughter of H and T, and was six years old. M is the son of H and G, and was 13 months old as of September 2013. The schematic view of the beluga pool is presented in Fig. 1. The beluga pool was composed of three sub-pools, which included a main pool (308 m^2^ surface area and 6.3 m deep), a holding pool (52 m^2^ surface area and 5.1 m deep), and a medical pool (53 m^2^ surface area and 5.0 m deep). The three pools were separated by metal lattices, but were acoustically linked. Each of the three belugas, excluding G and M, were isolated alone in the medical pool for experiments. G and M were not individually separated because of lactation, so they were segregated together in the medical pool. In May, G and M were separated because of reproductive reasons involving the three adults, and M and N were segregated together at that time. There were thus five isolation patterns: “H,” “T,” “N,” “G & M,” and “N & M.” Recordings were made between 08:00 and 18:00, excluding feeding and training times. A total of 46 sessions were conducted and each recording session continued for 30 min, with the exception of two 20-min sessions (Table 1). This study complied with the “WAZA Ethical Guidelines for the Conduct of Research on Animals by Zoo and Aquariums.” Research permission for this study was granted by the Port of Nagoya Public Aquarium, Japan. This study was observational study with temporal isolation of each individual, and our observation did not affect the whales’ welfare.

**Table 1:** Number of sessions and total recording time for each isolation pattern

Isolation pattern	H	T	N	G&M	N&M
Total recording time [min.]	560	180	270	290	60
(Number of sessions)	(19)	(6)	(9)	(10)	(2)

### Data collection

Acoustic recordings were taken using two TC4013 hydrophones (Reson Inc., Denmark), which were covered by PVC (polyvinyl chloride) pipes. The right and left hydrophones were then placed at a depth of 1 m in the medical pool and spaced 7.5 m apart from each other (Fig. 1). These hydrophones have a flat frequency response from 1 Hz to 170 kHz (−211 dB ±3 dB re 1 μPa/V at 1 m). The sound from the hydrophones was analog bandpass filtered from 1 kHz to 200 kHz, and it was amplified by 50 dB using an Aquafeeler III preamplifier (AquaSound Inc., Japan) with a flat frequency response to 200 kHz (+0/–3 dB). The output of the preamplifier was connected to two separate channels of an EZ7510 data recorder (NF Co., Japan), which digitized sounds from each channel sampling at 500 kHz and 16-bits. This recording system therefore had a relatively flat frequency response up to 170 kHz. Observations were made from an underwater window of the medical pool using an iVIS HF R11 video camera (Canon Inc., Japan).

### Sound categorization

The categorization of calls was used to estimate the call type that was most frequently produced in isolation. Belugas produce various complex calls. Previous studies tend to classify using narrow categories, but those categories differed among studies [32–42].

**Fig. 2:**
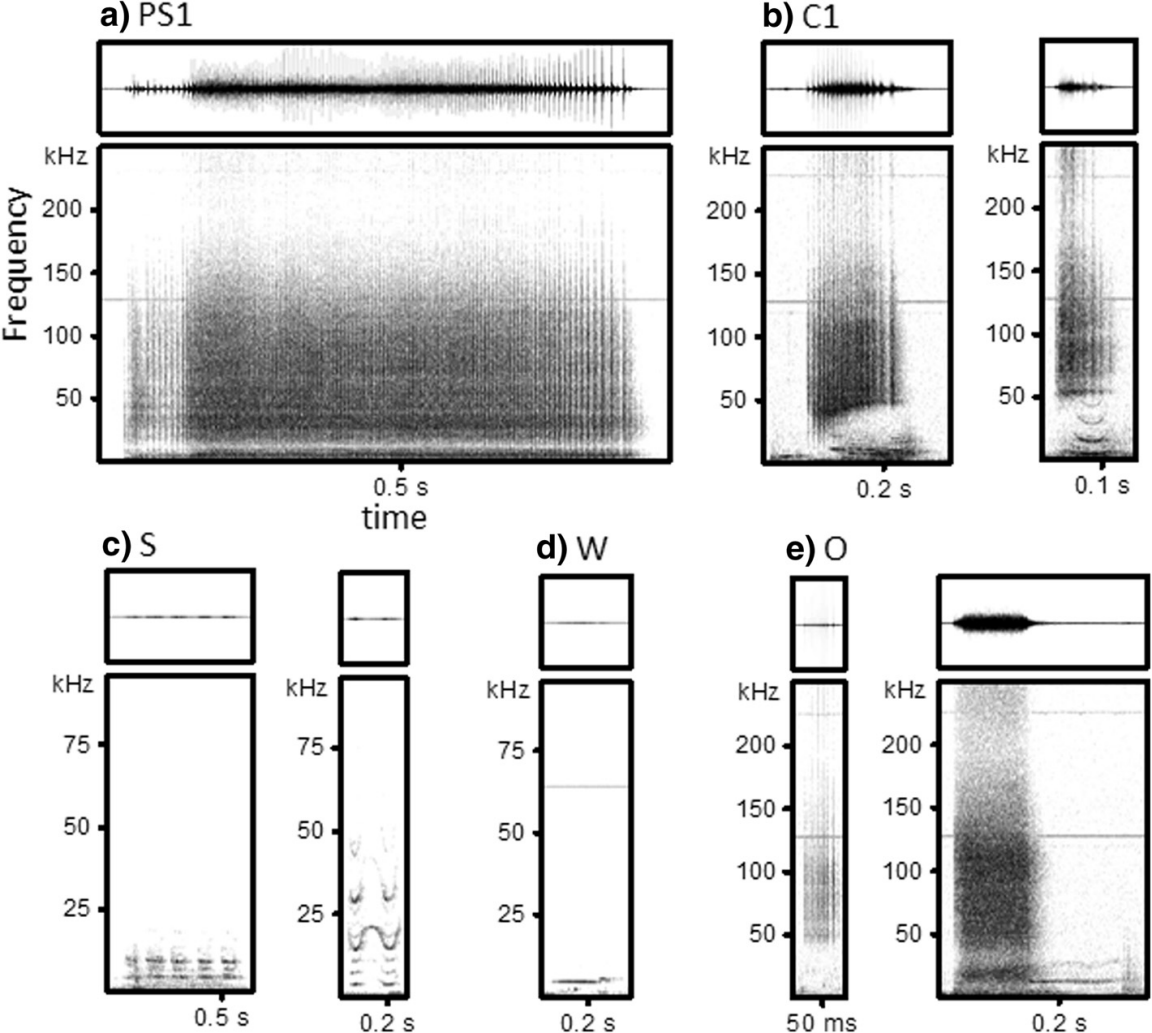
Examples of categorized call types: **a** PS1, **b** C1, **c** S, **d** W, and **e** O. The vertical and horizontal scales vary among call types

In the present study, all of the calls, except echolocation clicks that have IPIs generally longer than 20 ms and have high directivity, were divided into five broader categories. One of the authors (Y. Mishima) classified the calls based on visual and aural inspection using Audacity version 2.0.5 (The Audacity Team). Spectrograms were generated with a Hamming window function and FFT size of 1024. We identified four commonly produced call types: a) one type of pulsed call “PS1,” b) one type of combined call “C1,” c) short calls “S,” and d) whistles “W.” The remaining calls were classed into e) the “others” category (Fig. 2). The acoustical definition of each call type was as follows:

- PS1: The fixed pulse train, which sounds like a ratchet or a door creaking to human ears, is easy for humans to discriminate as PS1 calls [43]. Energy distributes broadband from less than 1 kHz up to at least 170 kHz, and this call continues for more than 150 ms. We added a type of combined call, comprising a mixture of pulse and tonal components, irregularly to this category. The pulse components resemble PS1 calls, and the tonal components overlap in both temporal and frequency domains.
- C1: This category includes combined calls that consist of two components. One component includes high-frequency broadband pulsed calls, and the other includes low-frequency narrowband tonal calls or low-frequency narrowband pulsed calls with different pulse repetition rates. The two components occur concurrently, but their frequency components do not overlap.
- S: Short calls. Low-frequency and tonal calls with and without some discrete harmonics. The duration is less than 150 ms, and some calls are repeated within 100 ms.
- W: Whistles. Low-frequency and tonal calls with and without some discrete harmonics. The duration is more than 150 ms.
- O: Others. This group includes burst pulses other than PS1 calls such as spectrographically sideband structured pulses with high pulse repetition rates [44]. This category also includes combined calls other than C1 calls such as graded calls with transitions from pulses to whistles [45], and noisy calls.

### Sound source localization

Arrival time differences to the two hydrophones were calculated using custom written MATLAB software to discriminate between the PS1 calls from isolated and non-isolated animals. The onset of PS1 was determined using a threshold that was nearly three times greater than the noise level. PS1 calls of the calf M frequently co-occurred with bubble streams, which is similar to the whistles of bottlenose dolphin calves [46]. Therefore, bubble streams were also used to identify PS1 calls from M in G & M and N & M isolation events. In cases where there was ambiguity in identifying whether the production was that of an isolated animal, the call was counted but excluded from the isolated individual calls.

### Analysis of PS1 calls

If at least one of the PS1 calls from the two hydrophones had a good signal-to-noise ratio, the PS1 call was further analyzed using Avisoft SASLab Pro version 5.2 (Avisoft Bioacoustics, Germany), and the call with the best signal-to-noise ratio of the two sets of data recorded by the hydrophones was used in the analyses. Pulses within the PS1 calls were automatically detected by Pulse Train Analysis in the software; however, we corrected some miscounted pulses manually in cases in which reflecting pulses had been counted or where direct pulses with lower amplitude had not been counted. Statistical analyses were performed using R software version 3.1.0 (The R Foundation for Statistical Computing).

Inter-pulse intervals (IPIs), duration, the number of pulses, and the pulse repetition rate (PRR) were extracted to determine individual differences. IPIs were the time differences from the peak of the preceding pulse to that of the following pulse. Changes in IPIs as a function of time, namely IPI contours, were depicted and compared visually. We calculated two average IPIs using the data for pulse nos. 11–20 and pulse nos. 11–20 from the last. The duration of PS1 indicated the time difference from the peak of the first pulse to that of the end pulse. The PRR was calculated by dividing the number of pulses by the duration. Univariate statistical analysis (Kruskal-Wallis test) was used on these temporal parameters to examine individual differences.

By using the pulses that composed the PS1 calls, power spectra were calculated for spectral analysis at three pulse locations, the third pulse, the middle pulse, and the third pulse from the last. The power spectra of pulses were quantified for the 1.5 ms of data containing each direct pulse, and were calculated with a Hamming window function and FFT size of 256 (frequency resolution was 1953 Hz and time resolution was 0.512 ms). The spectra were then smoothed using a five-point window. The analysis of noise spectra calculated using non-call windows before the onset of the PS1 calls revealed that the noise level below 5 kHz was consistently high and affected the PS1 spectra. The frequency range from 6 kHz to 170 kHz was therefore used for analyses, and the maximum source level (SL) in the range was set at zero to compare relative values. We calculated four parameters, peak frequency, the 10 dB bandwidth (i.e., the frequency band at a level of −10 dB from the peak; 10 dB BW), and the lower and upper frequencies of the 10 dB BW. First, the relative power spectra at each pulse location were averaged, and the averaged spectra among the three pulse locations for each animal were compared using the parameters to investigate whether there were spectral differences among the pulse locations within PS1. Next, the power spectra of the middle pulses were compared among individuals by visual inspection and by using univariate statistical analyses (one-way ANOVA) on the four spectral parameters.

Potential for individual coding (PIC), which was the ratio of inter-individual variation compared to intra-individual variation, was calculated to identify the temporal and spectral parameters that may encode individuality [47]. The inter- and intra-individual variations of each acoustic parameter were calculated using the coefficient of variation (CV). CV between individuals (CVb) was calculated according to the formula: *CVb* = (*SD*/*X*) * 100, where *SD* is the standard deviation and *X* is the average calculated for the overall sample. CV within individuals (CVw) was calculated according to the formula for small samples: *CVw* = (*SD*/*X*)(1 + 1/(4*n*)) * 100, where *SD* is the standard deviation, *X* is the average, and *n* is the sample size for an individual. The PIC value was calculated as follows: *PIC* = *CVb*/*mean CVw* where the *mean CVw* is the average value of the CVw for all individuals. Acoustic parameters with PIC >1 suggests that these parameters may be useful cues for individual recognition.

Discriminant function analysis (DFA) was then performed to classify PS1 calls into individuals based on acoustic parameters. However, before running DFA, we examined multicollinearity, multivariate outliers, multivariate normality, and homogeneity of variance-covariance matrices. The nine parameters, two average IPIs, duration, the number of pulses, PRR, peak frequencies, 10 dB BW, and the lower and upper frequencies of the 10 dB BW, were investigated. The variance inflation factors (VIF) of the nine parameters were measured using the “vif” function in the R package car to detect multicollinearity [48]. The number of pulses, the 10 dB BW, and the lower and upper frequency of the 10 dB BW had a high score with VIF >20.0. Excluding them from the data set, the remaining five parameters had VIF <2.0 and multicollinearity was not found; these were then included in the DFA. Potential multivariate outliers were searched for using robust Mahalanobis distances [49]. Because all of the PS1 calls from H and most of the PS1 calls from M were regarded as outliers, we included outliers in the analysis, with the exception of the outstanding one from T (see Additional file 1). Our data set did not satisfy multivariate normality and homogeneity of variance-covariance matrices (Shapiro-Wilk test, *P* < 0.0001 and Box’s M test, *P* < 0.0001, respectively); therefore, we performed quadratic DFA using the “qda” function in the R package MASS [50]. The five variables were entered into quadratic DFA and prior probabilities were set to equal sample size. The performance of quadratic DFA was quantified using a jack-knife leave-one-out cross-validation. Stepwise DFA using the “stepclass” function in the R package klaR was also performed to find the most informative parameters [51].

## Results

### Frequency of PS1 calls

A total of 6817 calls were recorded from both the isolated and the remaining animals in 46 isolation events over 22 h and 40 min. They were classified into 2633 PS1 calls, 1202 C1 calls, 793 S calls, 628 W calls, and 1561 others. PS1 was the most common call type, and it accounted for 38 % of the total calls followed by 18 % of S, 12 % of C1, and 9 % of W.

**Fig. 3:**
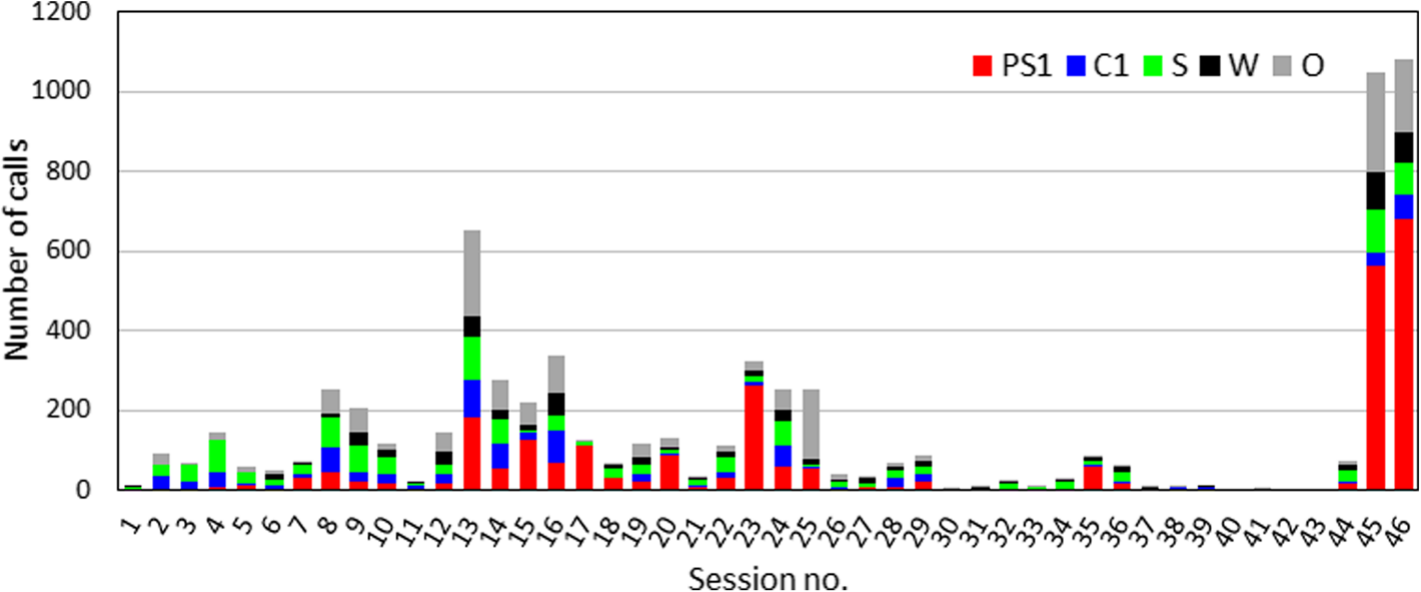
Frequency of each call type per session

The number of each call type was variable among sessions (Fig. 3). PS1 did not occupy the highest percentage during every separation, and in some sessions, PS1 did not occur. The highest call numbers were seen in sessions no. 45 and 46, and these correspond to N & M isolation events in May when the G-M (mother-calf) pair was separated. If the last two sessions are excluded, the percentages of PS1, C1, S, W, and O were 30, 15, 21, 10, and 24 %, respectively, and PS1 remained the major call type.

### Individual difference of PS1 calls

**Fig. 4:**
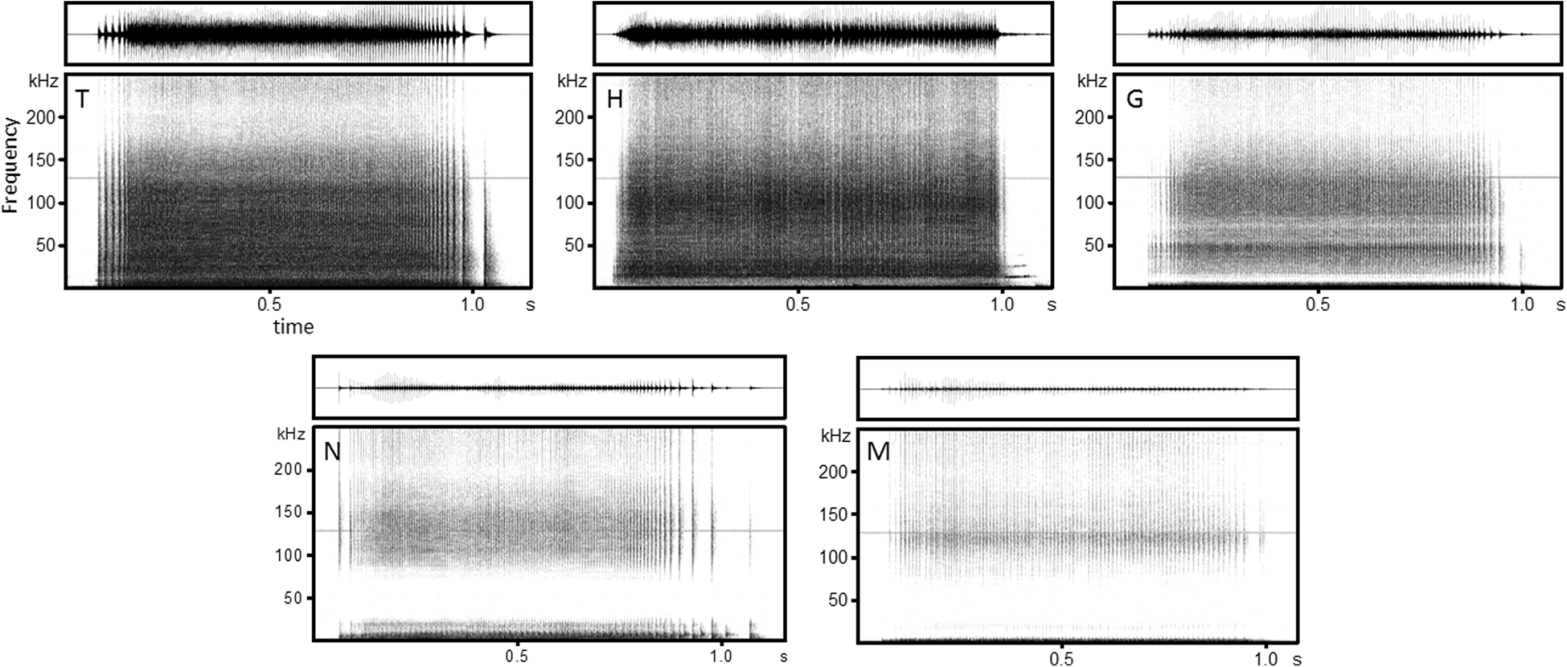
Examples of PS1 calls from five belugas. The upper figures show waveforms, and the lower figures show spectrograms. Individual identities are represented at the upper left in the spectrograms

Of the 2633 PS1 calls, 647 calls were produced by isolated animals whose calls could be discriminated, including 24, 331, 56, 80, and 156 by H, T, G, N, and M, respectively. The adult T produced a high number of PS1 calls, and she had the least number of isolation events. Spectrograms of individual PS1 calls are presented in Fig. 4. Most of the PS1 calls from the adult male H contained tonal components at around 13 kHz, and this structure was not found in any PS1 calls from the three females or the male calf.

**Fig. 5:**
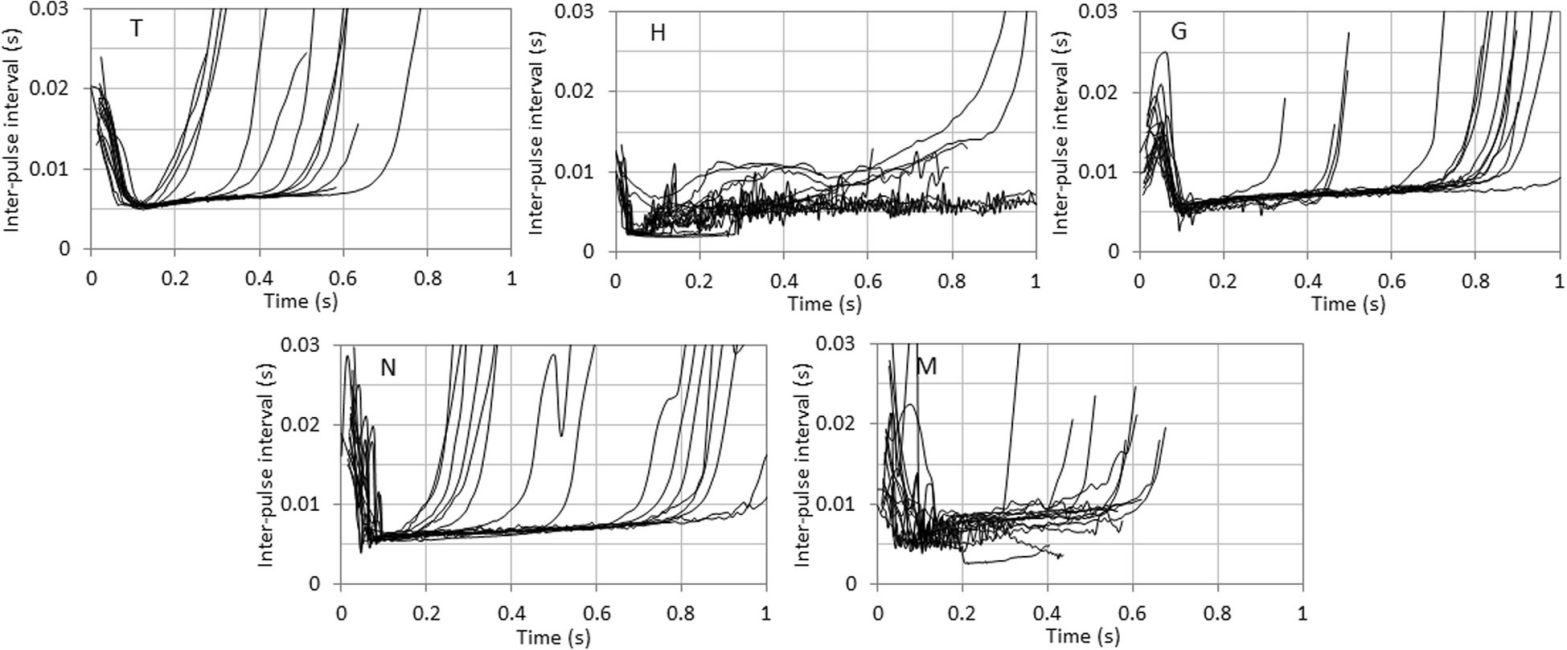
IPI contours of PS1 calls from five belugas (*n* = 16). Individual identities are represented at the *upper left*. Sixteen samples each from T, G, N, and M were randomly selected to match the number of depicted IPI contours of H from which the smallest samples were collected

Of the identified PS1 calls from isolated animals, 187 were used for further analysis, including 16, 97, 20, 21, and 33 for H, T, G, N, and M, respectively. IPI contours were depicted and compared among individuals (Fig. 5). Four belugas, excluding calf M, exhibited individually unique IPI contours (stereotyped IPI contours). The male H had lower stereotyped IPIs at the beginning, and his IPI contours were easily discriminated from others. In contrast, the three females (T, G, and N) had similar stereotyped IPI contours, but they contained slight differences at the beginning. The IPIs of T and G decreased at about the same time from the initial pulses. However, the slopes of IPI contours in T were gentler than that of G. In contrast, the slopes of IPI contours in T and N were similar, but a decrease in the IPIs of N occurred more rapidly from the initial pulses than that of T. The IPIs of PS1 calls from the calf M fluctuated highly over the duration.

Other temporal acoustic characteristics of PS1 for each beluga are shown in Table 2. A Kruskal-Wallis test revealed that the average IPI of pulse nos. 11–20, the average IPI of pulse nos. 11–20 from the last, the number of pulses, and the PRR were significantly different among individuals (χ^2^ = 76.65, *P* < 0.0001; χ^2^ = 26.20, *P* < 0.0001; χ^2^ = 26.21, *P* < 0.0001; and χ^2^ = 45.30, *P* < 0.0001, respectively), but duration did not differ significantly (χ^2^ = 4.73, *P* = 0.316).

**Table 2:** Temporal characteristics of PS1 calls from five belugas

ID	Number	Average IPIs of pulse nos. 11–20 (ms)			Average IPIs of pulse nos. 11–20 from the last (ms)			Duration (s)			Number of pulses			PRR (pulses/s)		
		Mean (sd)	Max	Min	Mean (sd)	Max	Min	Mean (sd)	Max	Min	Mean (sd)	Max	Min	Mean (sd)	Max	Min
H	16	2.92 (1.32)	6.96	2.05	7.76 (2.38)	13.06	5.67	0.77 (0.23)	1.13	0.36	144.2 (48.4)	234	64	190.8 (41.8)	248.9	90.1
T	97	5.69 (1.55)	19.58	4.48	7.12 (0.61)	8.42	4.91	0.76 (0.24)	1.48	0.25	83.5 (31.9)	154	19	110.1 (27.7)	220.9	32.6
G	20	5.67 (0.40)	6.4	4.92	7.91 (0.86)	8.93	5.9	0.80 (0.22)	1.18	0.36	100.5 (23.0)	145	46	128.3 (11.3)	152.4	111.2
N	21	5.96 (0.25)	6.53	5.48	7.6 (0.98)	9.38	6.14	0.79 (0.36)	1.36	0.29	82.6 (40.3)	144	32	104.1 (15.3)	137	68.2
M	33	6.85 (1.18)	10.8	4.96	8.24 (1.61)	11.07	4.42	0.68 (0.18)	1.13	0.36	80.2 (26.0)	180	43	119.7 (25.8)	182.1	40.9

Average IPIs of pulse nos. 11–20, average IPIs of pulse nos. 11–20 from the last, duration, the number of pulses, and the pulse rate are represented. Average, standard deviation, maximum, and minimum values were calculated for each parameter

Power spectra were calculated at three pulse locations along the third pulse, the middle pulse, and the third pulse from the last for each PS1, and were averaged at each location. Because all belugas showed similar patterns with an energy peak at 6 kHz in the averaged power spectra below 10 kHz, we used spectral parameters calculated in the range 11–170 kHz for individual comparisons (Table 3). No individuals exhibited high degrees of differences in peak frequency and the 10 dB BW depending on pulse locations. We thus selected the middle pulse as representative of the pulses comprising PS1.

**Table 3:** Spectral characteristics of PS1 calls from five belugas

ID		H	T	G	N	M
Number		16	97	20	21	33
Peak freq. (kHz)	3rd	109	29	113	109	115
	Middle	113	29	117	107	117
	3rd from last	109	29	115	109	117
10 dB BW (kHz)	3rd	133 [14–147]	113 [12–125]	72 [76–148]	88 [70–158]	72 [80–152]
	Middle	126 [22–148]	111 [12–123]	70 [78–148]	82 [72–154]	64 [86–150]
	3rd from last	119 [22–141]	74 [12–86]	72 [76–148]	88 [68–156]	64 [82–146]

In the frequency range 11–170 kHz, peak frequency and the 10 dB BW of averaged power spectra are represented for each of the three pulses (the third pulse, the middle pulse, and the third pulse from the last) out of those composing PS1. Values in brackets of the 10 dB BW represent the lower and upper frequencies of the 10 dB BW

**Fig. 6:**
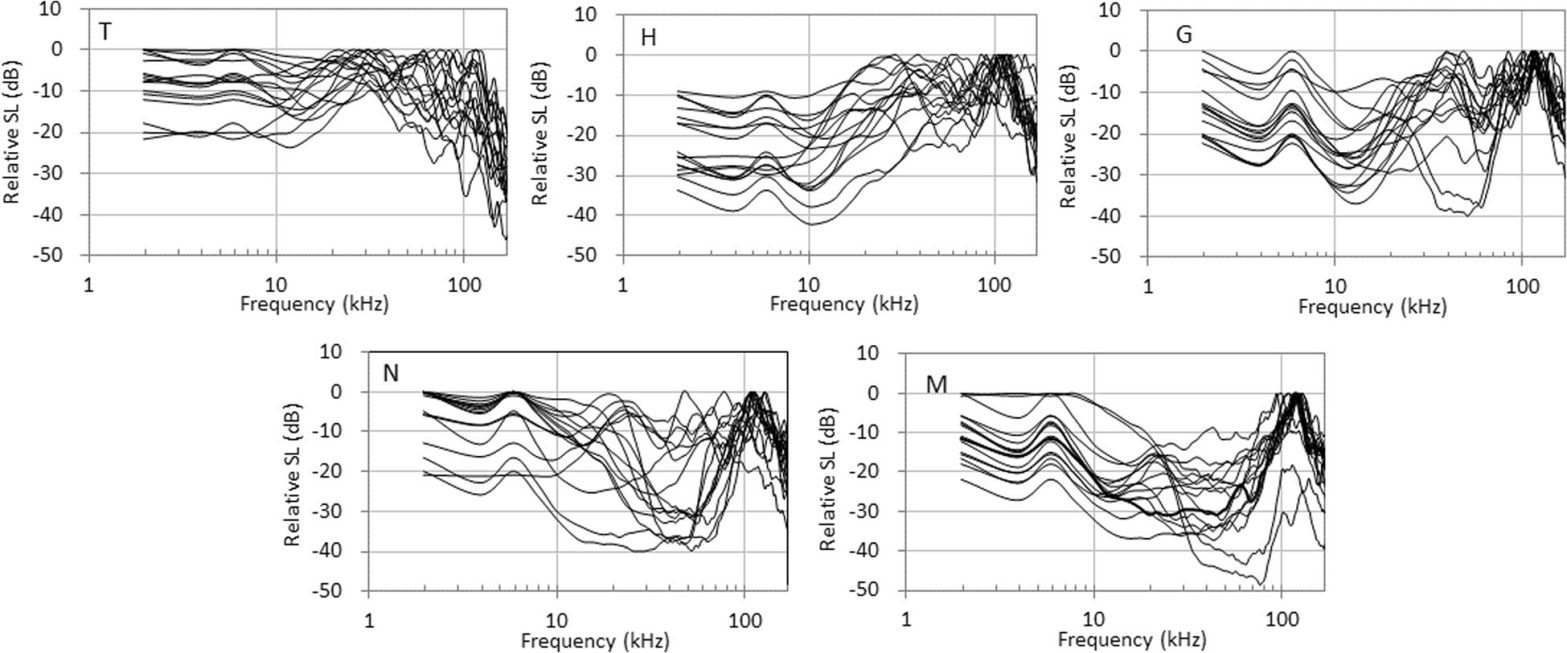
Power spectra of PS1 calls from five belugas (*n* = 16). Individual identities are represented at the *lower left*. The power spectra were calculated from the middle pulse location within PS1 calls. Sixteen samples each from T, G, N, and M were randomly selected to agree with the number of depicted IPI contours of H from which the smallest samples were collected

The power spectra of the middle pulses are shown in Fig. 6. Visual comparisons suggested that, unlike IPI contours, the power spectra of each beluga did not have apparent individual distinctiveness and stereotypy. A one-way ANOVA revealed that the peak frequency in the range 11–170 kHz was significantly different among individuals (*F* (4,182) = 37.87, *P* < 0.0001). Although the 10 dB BW did not differ significantly (*F* (4,182) =0.555, *P* = 0.696), its lower and upper frequencies were significantly different (*F* (4,182) = 45.10, *P* < 0.0001 and *F* (4,182) = 23.01, *P* < 0.0001, respectively).

We obtained PIC >1 for all temporal and spectral parameters, excluding the duration and the average IPI of pulse nos. 11–20 from the last (Table 4). The average IPI of pulse nos. 11–20, the PRR, the peak frequency, and the lower and upper frequency of the 10 dB BW had high PIC values; therefore these acoustic cues were more variable among individuals than within an individual.

**Table 4:** CVb, mean CVw, and PIC values calculated for each acoustic parameter

	Average IPIs of pulse nos. 11–20	Average IPIs of pulse nos. 11–20 from the last	Duration	Number of pulses	PRR	Peak freq.	Lower freq. of the 10 dB BW	Upper freq. of the 10 dB BW	10 dB BW
CVb	28.18	16.37	32.76	41.58	29.13	57.01	74.64	40.83	49.36
Mean CVw	20.41	16.66	32.66	35.52	18.60	38.54	51.59	29.52	45.83
PIC	1.38	0.98	1.00	1.17	1.57	1.48	1.45	1.38	1.08

The samples from T were reduced to 33, which was the same number used for M, and was selected randomly to reduce disparity in sample size (range 16–33) and increase the effectiveness of the quadratic DFA. The quadratic DFA based on the five variables, the average IPIs of pulse nos. 11–20, the average IPIs of pulse nos. 11–20 from the last, duration, PRR, and the peak frequencies, resulted in an overall correct classification rate of 80.5 %. The most powerful discriminator shown by stepwise DFA was the average IPIs of pulse nos. 11–20 followed by peak frequency.

## Discussion

PS1 was the predominant call type in isolation, and it accounted for 38 % of the total calls in all sessions. The PS1 call rate was highly variable among sessions, and some sessions had no PS1 calls (Fig. 3). Janik & Slater (1998) stated that, “Signature whistles were the most common whistles in the isolation context but did not occur during every separation” [10]. The PS1 call rate might be associated with many factors, such as activity state, presence of trainers at the poolside, and habituation for separation similar to the signature whistles of bottlenose dolphins [52]. The subjects, excluding the calf, were practically trained to be isolated in the experimental pool, and they possibly may have become accustomed to being separated from the group. The time from separation to the start of the recordings varied among sessions, and it also appeared to cause different degrees of habituation. Several factors might influence their motivational state, and may have introduced variability to PS1 call rates. The remarkably high number of calls in sessions no. 45 and 46 (N & M isolation events) was likely due to the separation of the G-M (mother-calf) pair (Fig. 3). Although the calf only produced three PS1 calls in all of the G & M sessions when segregated with his mother, he dramatically produced PS1 calls at the metal lattice once he was separated from his mother. This was a strong indication that PS1 calls served as isolation calls. This possibility was supported by Vergara et al. (2010), which reported a high number of mother-calf contact calls in isolation context [39]. G and M exchanged PS1 calls frequently to contact each other, and this might have activated the utterances of other pool mates. However, we could not rule out the possibility that the high call rate was associated with the reproductive season. The recordings were conducted in May when the three adults were reproductively active, and they produced various sounds.

The considerably high PS1 call rate of T might be related to temperament and social rank. T had a strong character, and was the oldest and largest female. Although dominance was not specifically measured, she appeared to be top-ranked based on previously utilized criteria [33]. There is also the possibility that active and dominant belugas produce contact calls at higher rates.

Only PS1 calls from the adult male H contained tonal components at around 13 kHz (Fig. 4). Of the five Type A call variants (A1–A5) described by Vergara et al. (2010), these calls are similar to A1, which was produced by an adult female and her two offspring [39]. A1 has an average PRR of 94.6 pulses/s, 1.2–1.9 s in duration, and it consistently contains a narrowband tonal component at 14.6 kHz. The A4 call overlaps with A1 in PRR and duration. However, A4 lacks the tonal component, and is similar to PS1 calls produced by individuals other than the male H in this study. Therefore, some of the Type A calls may be considered PS1 calls. The “combined tonal/pulsed signals” of narwhals (*Monodon monoceros*), which belong to the Monodontidae family, which also includes belugas, have a similar acoustical structure [53–55]. They possibly use the combined tonal/pulsed signals as individualized contact calls since the pulse-rate contours of the pulse components were clearly different between two adult males [54]. A larger sample size is needed to determine the function of the common tonal components in PS1 calls, Type A1 calls, and the combined tonal/pulsed signals.

Some acoustic parameters in PS1 calls differed inter-individually. For instance, H had clearly distinctive IPI contours, and T, G, and N had similar, but slightly different, IPI contours at the initial part (Fig. 5). They might possibly recognize these slight temporal differences, because belugas have a high temporal resolution of around 1400 Hz in cut-off frequency [56]. Similar types of information media were present in the contact calls of narwhals and resident killer whales (*Orcinus orca*). As described above, two narwhals had clearly different pulse-rate contours in the combined tonal/pulsed signals [54]. Killer whales share group specific pulse-type contact calls that are designated as “discrete calls” among group members, and they use pulse-rate contours in discrete calls for group information [57, 58]. To confirm whether IPI contours of PS1 calls are specific to individuals, we should perform human observer classification [59] and establish an automated classification method in reference to the signature whistles of bottlenose dolphins and the discrete calls of killer whales [60].

The initial part of IPI contours was individually distinctive (Fig. 5) and it was supported by the PIC results showing that the average IPI of pulse nos. 11–20 had PIC >1 but the average IPI of pulse nos. 11–20 from the last had PIC <1 (Table 4). It is not surprising that the initial part of the PS1 calls transmit individual information. Calls in 88 % of PS1 exchanges by two different individuals occurred within 1 s of each other [43]. Furthermore, most of those were overlapping exchanges, or the second caller responded before the termination of the first caller’s PS1. This suggests that belugas recognize and respond to the individually distinctive initial part of PS1 calls. Similarly, Vergara et al. (2010) found that the broadband Type A calls they described were used in antiphonal call matching exchanges, and they also contained overlapping exchanges [39].

The calf, M (aged 21 months in N & M session nos. 46 and 47 when several PS1 calls were collected from M) produced temporally fluctuating PS1 calls (Fig. 5), and they co-occurred with bubble streams. It was inferred that he was still in the course of vocal production learning [15] or morphological development. The following facts support this hypothesis: 1) a male beluga calf developed the same type of pulsed contact call described here until it was at least 1 year old, and the vocal development process continued past his first year of life [28]; 2) wild beluga calves stay with their mothers until they are at least 3 years old [61]; 3) captive male belugas reach maturity at nine years old or older [62, 63]; and 4) the 6-year-old sub-adult female N (captive females reach maturity at five years old or older [62, 63]) produced highly stereotyped PS1 calls. If these assumptions are true, belugas may develop stereotyped individual calls later than bottlenose dolphins, which develop them during the first year life [8].

Univariate statistical analyses revealed that inter-individual differences existed in the two average IPIs, the number of pulses, and the PRR; however, duration did not differ significantly. The PIC measure also showed that duration did not have inter-individual greater than intra-individual variability (Table 4). Similarly, duration was not stereotyped in the signature whistles. In the signature whistles of bottlenose dolphins, each of the repeated basic contours is called a loop. Loop number, loop duration, and the inter-loop interval, which are related to whistle duration, were affected by motivational state [52]. Indo-Pacific bottlenose dolphins (*Tursiops aduncus*) also showed a high degree of variation in the duration of each signature whistle type [64]. Thus, duration appears to lack individual distinctiveness in both pulse-type and whistle-type contact calls.

In each beluga, the third pulse, the middle pulse, and the third pulse from the last of the PS1 calls had similar power spectra (Table 3). This suggests that the frequency characteristics of pulses were relatively fixed within PS1 calls regardless of pulse location. Thus, belugas might not use change in frequency characteristics within PS1 calls for individual information.

Individual differences were not found visually in the power spectra of middle pulses, especially below 10 kHz (Fig. 6). Information in the frequency band from 10 to 110 kHz may be effectively used by belugas in captivity because they have high hearing sensitivity in that range [56, 65]. Univariate statistical analyses revealed that the peak frequency and the lower and upper frequencies of the 10 dB BW had individual differences in the frequency range. In addition, all of the parameters had inter-individual greater than intra-individual variability with PIC >1 (Table 4). However, visual inspection of power spectra in the frequency range, unlike IPI contours, did not uncover apparent individual specificity, and found variability in the intra-individual spectra (Fig. 6). The results of these visual investigations may appear counter-intuitive, as human observers have proven to perform better than computers at classifying calls [59]. Directivity may have affected the intra-individual differences, as the angle between the animals’ heads and the hydrophones was not taken into account in calculating individual spectra. In the case of broadband echolocation clicks produced by belugas, the beam pattern was highly directional with a -3 dB beamwidth of 6.5° in the horizontal and vertical planes [66]. The sound field of clicks varied in accordance with frequency [67]. In addition, dolphins can steer beams of clicks [68]. Although the directivity of communicative pulsed sounds in cetacean was not studied, it appeared to be lower than that of the echolocation clicks used to broadcast the caller’s information or message. In practice, power spectra tended to be similar between PS1 calls recorded on the two hydrophones. However, the possibility remains that directionality may explain some of the spectral variations. A wide frequency band tended to be missing for individuals N and M, and it might represent the fact that calls of N and M tended to have lower amplitude than those of adults.

Quadratic DFA classified PS1 calls into individuals with an overall correct classification rate of 80.5 %. Stepwise DFA showed that the most informative variables were the average IPIs of pulse nos. 11–20 and peak frequency in the range 11–170 kHz. It was unclear at this stage whether the temporal and spectral parameters related to signature or were by-products of voice feature attributed to difference in the body size or sex. However, given that the initial part of the IPI contours was individually stereotyped and the average IPI of the initial part was the most informative parameter in DFA, the pulse repetition pattern had a high possibility of being a signature function if belugas encoded signature information in contact calls. However, there was no apparent individual distinctiveness in power spectral shapes, especially below 10 kHz, and the spectral components of high frequency are unstable in the whales’ environment because they are affected by transmission loss; therefore, the spectra may be inappropriate as the carrier for the signature.

Broadband relatively long duration pulsed calls such as the variant PS1 were described in previous studies of both captive and wild beluga vocalizations [32–35, 38–42], sometimes in the same isolation context [35, 39]. It should be noted that previous studies only recorded up to 24 kHz or lower; therefore, frequency components were not compared directly in this study. Sjare and Smith (1986a) reported vocalizations of wild belugas in the Northwest Territories, Canada [32]. The pulsed vocalization type H they categorized in group 3 calls is similar to PS1 calls in terms of spectrograms and PRR of 80–290 pulses/s. The duration of the group 3 calls are also similar to PS1 calls, with 0.85 ± 0.44 (0.2–2.7) s. The frequency composition of the group 3 calls is 4.6 ± 1.7 (0.3–12.0) kHz, which is comparable to PS1 calls that have a peak at 6 kHz in the range below 10 kHz. The group 3 calls were produced during rest and socially interactive periods [69].

Belikov and Bel’kovich (2008) examined wild belugas in the White Sea, Russia [38]. The pulsed call type with low PRR, lPT3, and lPT7 resemble PS1 calls. The IPT3 had 13–630 pulses/s, 0.87 ± 0.43 (0.32–2.28) s in duration, and a dominant frequency of 6.1 ± 1.0 (3.9–8.7), while the IPT7 had 10–770 pulses/s, 1.36 ± 0.43 (0.6–2.43) s in duration, and a dominant frequency of 5.7 ± 4.8 (0.2–15.0). These calls were produced during social interactions and quiet swimming [40]. Alekseeva et al. (2013) reported that pulsed calls with low PRR, groaning and grumbling, which are similar to PS1 calls, were produced in the context of sexual behavior [42].

Karlsen et al. (2002) investigated vocalizations of wild belugas in Svalbard, Norway [34]. The pulsed call type ll is similar to PS1 calls, with PRR of 104 ± 64 (23–240) pulses/s, 0.55 ± 0.54 (0.07–3.12) s in duration, and frequency range of 0.2–20.0 kHz. The type ll calls were produced in the context of milling, travelling, and joining. Van Parijis et al. (2003) recorded calls from wild belugas in the same Svalbard area during temporal capture events [35]. In their recordings, the mother of a mother-calf pair produced click trains with an average of 27 pulses/s (0.012–0.46 s IPIs) and 1.9 ± 1.3 s in duration. She frequently moved her head toward her calf while producing sounds. The click trains from the calf had an average of 18 pulses/s (0.09–0.5 s IPIs) and 0.6 ± 0.5 s in duration. Another sub-adult female that was temporarily captured also produced click trains with an average of 22 pulses/s (0.03–0.41 s IPIs) and 0.3 ± 0.08 s in duration. These calls have a smaller PRR than PS1 calls but are similar in duration.

Chemelnitsky and Ferguson (2012) represented vocalizations of wild belugas in the Churchill River, Canada [41]. The pulsed call type P2 is similar to PS1 calls spectrographically. P2 has a PRR of 207 ± 57 pulses/s, 1.16 ± 0.36 s in duration, and frequency range of 2.8–5.3 kHz. Recchia (1994) reported on captive belugas originating from the river. One of the most discriminant call types, the so-called ‘buzzsaw’ calls, appears to be spectrographically similar to PS1 calls with a minimum duration of 0.2 s, but the information available is limited [33]. Vancouver Aquarium belugas originating from the Churchill River Estuary produced Type A calls in an isolation context, some of which are similar to the PS1 calls as described above [39].

Subsequent recordings and individual comparisons of PS1 calls from captive and wild beluga populations are essential to confirm whether those individual differences are common in belugas. As a further step, playback experiments are also needed to assess whether the differences in PS1 calls are perceived as individual differences by the belugas, and, if so, which parameter is used for individual recognition and whether it is a signature independent of voice cues. Additionally, a long-term study of the development and stability of PS1 calls should be conducted. Comparisons between PS1 calls in belugas and signature whistles in bottlenose dolphins will help us determine how contact calls evolved in cetaceans.

## Conclusion

The present study showed that the captive belugas most frequently produce individually distinctive PS1 calls in isolation. Given that the IPI contours of PS1 calls are individually stereotyped and the average IPI is the most informative parameter in stepwise DFA, the pulse repetition pattern has a high possibility of being a signature function if it encodes signature information in contact calls, as seen in bottlenose dolphins. Larger sample sizes and playback experiments may help to confirm whether PS1 calls in belugas are functionally similar to signature whistles in bottlenose dolphins.

## Additional file

Additional file 1:
**Outliers searched by robust Mahalanobis distances.** The samples are shown from the left in the order of H, T, G, N and M. Red circles represent the outliers. (PNG 61 kb)

## Abbreviations

PS1: One type of pulsed sound
C1: One type of combined call
S: Short calls
W: Whistles
O: Other calls
IPI: Inter-pulse interval
PRR: Pulse repetition rate
10 dB BW: 10 dB bandwidth
PIC: Potential for individual coding
CV: Coefficient of variation
CVb: Coefficient of variation between individuals
CVw: Coefficient of variation within individuals
DFA: Discriminant function analysis
VIF: Variance inflation factors
SL: Source level
