# Supplementary figures and images for: Individuality embedded in the isolation calls of captive beluga whales (Delphinapterus leucas)
Source: Zoological Lett. 2015 Oct 1;1:27. doi: 10.1186/s40851-015-0028-x (PMC4657357; doi:10.1186/s40851-015-0028-x)

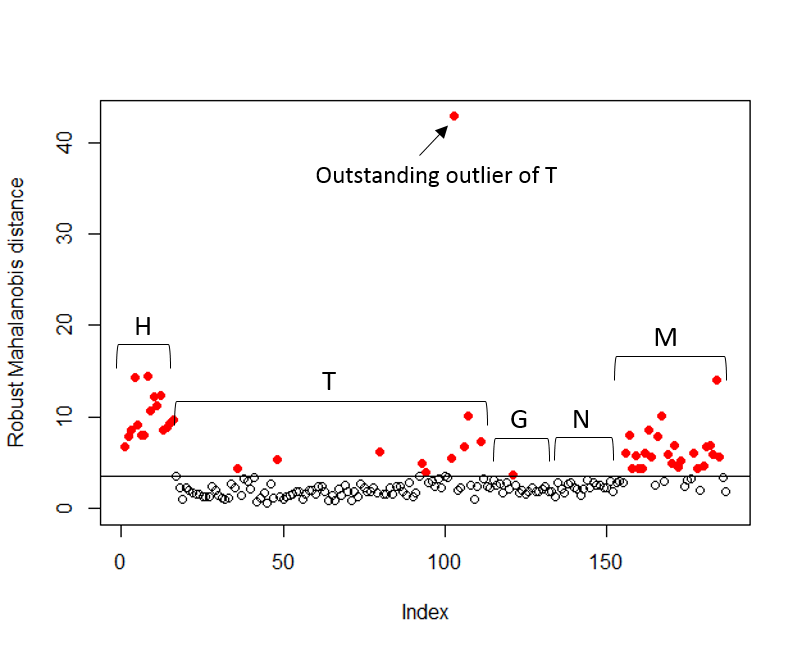

Supplement: Additional file 1: — Outliers searched by robust Mahalanobis distances. The samples are shown from the left in the order of H, T, G, N and M. Red circles represent the outliers. (PNG 61 kb) [file 40851_2015_28_MOESM1_ESM.png]
